# Cluster headache associated with acute maxillary sinusitis

**DOI:** 10.1186/2193-1801-2-509

**Published:** 2013-10-05

**Authors:** Bengt Edvardsson

**Affiliations:** Department of Neurology, Faculty of Medicine, Skane University Hospital, Lund, S-221 85 Sweden

**Keywords:** Cluster headache, Acute maxillary sinusitis, Secondary, Symptomatic, Infection

## Abstract

**Background:**

Cluster headache is a primary headache by definition not caused by any known underlying structural pathology. However, symptomatic cases have been described, for example tumours, particularly pituitary adenomas, malformations, and infections/inflammations. The evaluation of cluster headache is an issue unresolved.

**Case description:**

I present a case of a 24-year-old patient who presented with a 4-week history of side-locked attacks of pain located in the left orbit. He satisfied the revised International Classification of Headache Disorders criteria for cluster headache. His medical and family histories were unremarkable. There was no history of headache. A diagnosis of cluster headache was made. The patient responded to symptomatic treatment. Low-dose computer tomography scan after 2 weeks displayed a left-sided acute maxillary sinusitis. The headache attacks resolved completely after treatment with antibiotics and sinus puncture.

**Discussion and evaluation:**

Although I cannot exclude an unintentional comorbidity, in my opinion, the co-occurrence of an acute maxillary sinusitis with unilateral headache, in a hitherto headache-free man, points toward the fact that in this case the cluster headache was caused or triggered by the sinusitis. The headache attacks resolved completely after the treatment and the patient also remained headache free at the follow-up. The response of the headache to sumatriptan and other typical cluster headache medications does not exclude a secondary form. Symptomatic cluster headaches responsive to this therapy have been described. Associated cranial lesions such as infections have been reported in cluster headache patients and the attacks may be clinically indistinguishable from the primary form.

**Conclusions:**

Neuroimaging, preferably contrast-enhanced magnetic resonance imaging including sinuses should always be considered in patients with cluster headache despite normal neurological examination. Acute maxillary sinusitis can present as cluster headache.

## Background

Cluster headache (CH) is a primary headache, by definition not caused by any underlying structural pathology and belonging to the group of trigeminal-autonomic cephalalgias (Headache Subcommittee of the International Headache Society 2004). CH is the most frequent syndrome in this group. The characteristic symptoms are strictly unilateral head pain (mainly around orbital and temporal regions) and associated ipsilateral cranial autonomic features. The headache usually lasts 45 to 90 minutes, but can range between 15 and 180 minutes. A circannual and circadian pattern is typical. Symptomatic cases of CH have been described, for example tumours, particularly pituitary adenomas, malformations, and infections/inflammations (Cittadini & Matharu 2009). The question whether patients with CH should undergo neuroimaging to exclude a causal underlying structural lesion is unresolved. I here report a case of acute maxillary sinusitis the symptoms characteristics of which fully comply with the criteria of cluster headache (Headache Subcommittee of the International Headache Society 2004). Symptomatic CH due to maxillary sinusitis is rare. Previous cases have been described by Takeshima et al. (Headache 1998; 28:208–208) and Molins et al. (Med Clin (Barc) 1989; 92:181–183).

## Case description

**Figure 1:**
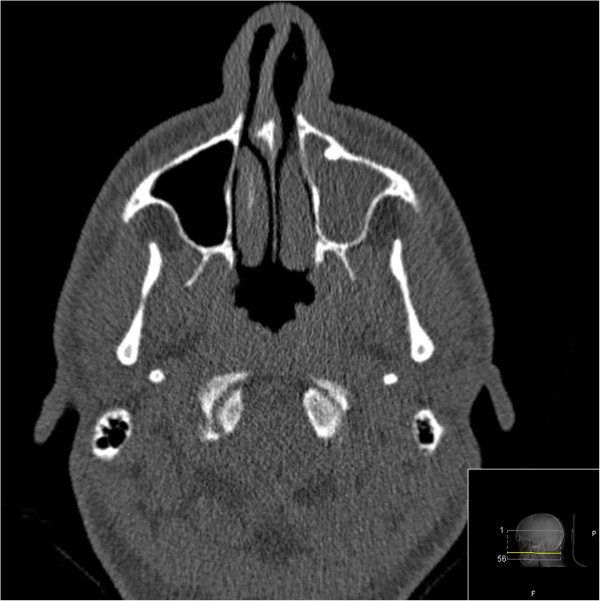
**Low-dose computer tomography scan, showing a left-sided acute maxillary sinusitis.**

**Figure 2:**
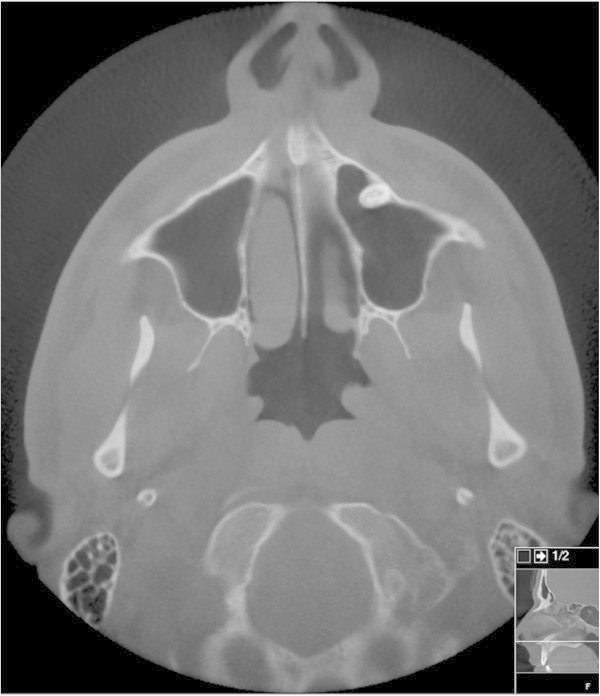
**Low-dose computer tomography scan 6 weeks later, showing normal findings.**

A 24-year-old man presented with a 4-week history of side-locked attacks of excruciatingly severe stabbing and boring left-sided pain located in the orbit. The attacks were associated with nasal obstruction, clear nasal discharge, conjunctival injection and restlessness. No continuous background pain was identified. The duration of the attacks was about 40 minutes and the frequency 3 per 24 hours, 5 days a week. There was no history of headache. His medical and family history was otherwise unremarkable. He was not on any medications and used no drugs. Vital signs, physical examination, and neurological examination were normal. Local tenderness over the sinuses was not found. Laboratory testing was normal. He satisfied the revised International Classification of Headache Disorders criteria for cluster headache. A diagnosis of CH was made and subcutaneous sumatriptan as well as oxygen 100% (7 L/min) were prescribed. The patient responded to subcutaneous sumatriptan/oxygen with relief within 20 minutes. A follow-up was planned. An otologist consultation was performed to rule out an acute sinusitis. Low-dose computer tomography scan after 2 weeks displayed a left-sided acute maxillary sinusitis (Figure 1). A sinus puncture was performed and it displayed acute inflammation/high leukocyte count. Bacterial culture displayed Haemophilus influenzae. The headache attacks resolved completely after treatment with antibiotics and sinus puncture. A low-dose computer tomography scan was repeated after 6 weeks and it displayed normal findings (Figure 2). The prescribed medication was discontinued. No additional treatment was given. He remained headache-free and had not experienced any headache attacks at follow-up after several years.

## Discussion

The case study highlights a patient with CH responding to treatment. Evaluation revealed an acute maxillary sinusitis. The patient satisfied the revised International Classification of Headache Disorders criteria for CH (Headache Subcommittee of the International Headache Society 2004). Although I cannot exclude an unintentional comorbidity, in my opinion, the co-occurrence of an acute maxillary sinusitis with unilateral headache, in a hitherto headache-free man, points towards the fact that in this case the CH was caused or triggered by the sinusitis. The headache attacks resolved completely after treatment and the patient also remained headache free at the follow-up after several years. An alternative explanation could be the following: during CH attacks autonomic symptoms, including nasal congestion, are commonly observed. Nasal congestion could predispose the patient to develop an acute sinusitis. A spontaneous remission of an episodic CH could be misinterpreted as being an effect of the antibiotic treatment. However, the patient remained free of CH attacks at the follow up after several years and had not previously suffered from CH.

The response of the headache to sumatriptan and other typical CH medications does not exclude a secondary form (Ad Hoc Committee on Classification of Headache 1962;Testa et al. 2008). Associated cranial lesions such as tumours have been reported in CH patients and the attacks may be clinically indistinguishable from the primary form (Ad Hoc Committee on Classification of Headache 1962;Favier et al. 2007). (Mainardi et al. 2010) identified 156 secondary cluster-like headache cases published from 1975 to 2008. They found in the review that vascular pathologies, for example intracranial aneurysms and dural fistulas were the first cause of secondary CH, followed by tumours and inflammatory/infectious diseases, the latter accounting for 13.1% of cases. Among the inflammatory/infectious cases, two cases were associated with sphenoidal aspergillosis and one each with ophthalmic herpes zoster, post infection from herpes simplex and maxillary sinusitis. The article also reports two cases of cluster-like headache (not fulfilling the criteria for CH) associated with sinusitis.

The pathophysiology of CH is not well known. The most widely accepted theory is that primary CH is characterized by hypothalamic activation with secondary activation of the trigeminal-autonomic reflex, probably by a trigeminal-hypothalamic pathway (Cittadini & Matharu 2009). The exact pathophysiology in this case is unknown. A structural lesion may cause autonomic imbalance, resulting in periodic fluctuations in the activity of the autonomic nervous system, ultimately leading to an attack-wise presentation of the symptoms (Wilbrink et al. 2009). Differences in the individual threshold for triggering the parasympathetic trigeminal reflexes may also play a role (Straube et al. 2007). The pain mechanism in secondary CHs seems ascribable to irritation of pain-sensitive structures and activation of trigeminal nerve endings (Leone & Bussone 2009).

Attempts have been made to define red flags indicating a secondary cause when cluster-like headache appears for the first time (Mainardi et al. 2010). The authors of that study emphasize in their report that, at first observation, 50% of patients with secondary CH presented as cases fulfilling the criteria for CH, perfectly mimicking CH. Therefore, the likelihood that a secondary cause is responsible for a clinical picture mimicking a primary CH, albeit low, should always be considered (Mainardi et al. 2010). This opinion is in accordance with the reviews by (Favier et al. 2007) and by, (Wilbrink et al. 2009) which recommend neuroimaging in all patients with trigeminal-autonomic cephalalgias. Magnetic resonance imaging is the preferred procedure for imaging in CH cases because of its greater sensitivity to vascular disease, tumour, demyelinating disease, and infections/inflammations (Wilbrink et al. 2009;Mainardi et al. 2010).

## Conclusions

CH might in rare cases be the presenting symptom of an acute maxillary sinusitis even in typical forms of that headache. Neuroimaging, preferably magnetic resonance imaging including sinuses should always be considered in patients with CH.

The author takes full responsibility for the data presented in this study, analysis of the data, conclusions, and conduct of the research. The author had full access to those data and has maintained the right to publish any and all data independent of any third party.

Concerning approval of human studies by the appropriate ethics committee and therefore performed in accordance with the ethical standards laid down in the1964 Declaration of Helsinki: In this case this is not appreciable.
